# Evaluation of equine perineural anesthesia simulators with integrated success control for veterinary education

**DOI:** 10.3389/fvets.2024.1403794

**Published:** 2025-01-09

**Authors:** Anna Chodzinski, Sandra Wissing, Andrea Tipold, Florian Geburek

**Affiliations:** ^1^Centre for E-Learning, Didactics and Educational Research, Clinical Skills Lab, University of Veterinary Medicine Hannover, Foundation, Hannover, Germany; ^2^Clinic for Small Animals, Department of Neurology, University of Veterinary Medicine Hannover, Foundation, Hannover, Germany; ^3^Clinic for Horses, Department of Surgery and Orthopedics, University of Veterinary Medicine Hannover, Foundation, Hannover, Germany

**Keywords:** teaching, Day One Competences, skills, orthopedics, horse, cadaver, limb, lameness

## Abstract

The skills necessary to perform diagnostic perineural anesthesia in equids belongs to one of the Day One Competences of a veterinarian, so every veterinary graduate should be able to perform them correctly. For logistical, hygienic and ethical reasons, practical exercises on cadaver limbs are not accessible to all students. Two equine distal limb simulators were developed and evaluated as an additional instructional tool to train the required skills. Both simulators were designed and built with an integrated success control, with Simulator I (S1) designed to be a simplified anatomical model and Simulator II (S2), a more realistic model. The simulators were tested by 68 students in the 5^th^ year who were divided into two groups. Thirty-four students received a training session using the simplified anatomical model (S1) and the other 34 students one on cadaver limbs, the usual instructional tool. The practical learning success of both groups was validated using S2. Additionally, data on self-efficacy were collected. The results show that the two groups did not differ significantly in their practical learning success, whereas self-efficacy of both groups increased significantly after the sessions. An evaluation performed by 7 veterinarians and 49 students of the 5^th^ year indicate that the simulators are suitable for teaching perineural anesthesia in the equine distal limb. However, S2 could be more realistic. The simulators will be used as a supplement to exercises on cadaver limbs to enable all students to practice perineural anesthesia.

## 1 Introduction

Perineural anesthesia is a vital diagnostic tool frequently used by equine veterinarians in horses with subacute to chronic lameness (1, 2). If clinical findings are not indicative of the cause of lameness and acute signs of inflammation have subsided, diagnostic anesthesia of peripheral nerves is the current standard to localize pain anatomically within the limb (2, 3). Consequently, the skills to perform diagnostic perineural anesthesia correctly are considered as Day One Competences of veterinarians (4). A small volume of local anesthetic is applied perineurally to peripheral nerves of the limb so that pain transmission is interrupted (1, 2). Thus, the anatomical region distal to the site of injection is anesthetized. Pain and thereby lameness caused by structures within the anesthetized region are temporarily eliminated (1, 2). Usually intrasynovial anesthesia and/or diagnostic imaging of the relevant region are applied next to specify a diagnosis (1).

Performing perineural anesthesia correctly requires profound anatomical knowledge of the correct puncture sites and the puncture technique itself, including properties of tissues that are penetrated (1, 5). Traditionally, cadaver limbs are used to practice punctures, and a dye such as methylene blue is injected instead of local anesthetic, which can be easily detected subcutaneously after dissection to verify proximity to the nerve (5). However, due to logistical, hygienic and ethical reasons, the opportunity to practice on cadavers is limited for students, so that an increasing number of simulators and models have been introduced for practical training at veterinary faculties and educational institutions. Several studies already demonstrated that simulators are suitable for practical training in veterinary medicine (6–8). Besides the logistical, hygienic and ethical advantages, feedback can be obtained repeatedly as often as desired on simulators, and repetition is considered as important *per se* while adapting practical skills (9, 10). In addition, simulation-based learning has a positive impact on students' self-efficacy (6, 7, 11–13). A high self-efficacy motivates students to deal with difficult tasks and to develop solution strategies (14, 15) and is advantageous when working on live animals (16). In order to maintain a learning effect in simulation-based training, feedback is essential. Mahmood and Darzi (17) described that training on a colonoscopy simulator in the absence of feedback is not successful. In addition to subjective feedback from tutors, auto-feedback can be used and learners receive immediate objective feedback from the simulator itself. An example of this is a venipuncture trainer, which provides feedback about puncturing the vein via the leakage of fluid through the puncture needle (7).

At the University of Veterinary Medicine Hannover, Foundation (TiHo), Hannover, Germany, perineural anesthesia is currently taught on cadaver limbs. Due to the reasons mentioned above, it is not possible for every student to practice or repeat the skill numerous times. Therefore, exercises on cadaver limbs will be supplemented by exercises on simulators in the future. The existing commercially available simulator “Equine Distal Limb Nerve Block” (Holsim, Hamilton, New Zealand) (18) and the simulator developed at the Royal Veterinary College, London (8) integrate puncture sites up to and including the Low four-point nerve block. In this study, this should be extended proximally to include the High four-point nerve block. In this way, a complete examination of the equine distal limb can be practiced with the help of perineural anesthesia where the most common causes of lameness in horses are localized (19, 20). In addition, the realistic model should also allow the application of fluid to ensure a more realistic performance of perineural anesthesia. The ideas described by Gunning et al. (8) were incorporated in the development of the simulators.

The current study was conducted at the Clinical Skills Lab (CSL) in collaboration with the Clinic for Horses of the TiHo.

The aim of this study was to develop two simulators for practical training of perineural anesthesia in the distal limb of horses up to the high four-point nerve block as taught at the TiHo, and to provide feedback when a puncture has been performed correctly by means of an integrated success control. One of the simulators was a simplified anatomical model, while the second simulator was a more realistic representation of the equine distal limb. The simulators were evaluated for their realism and suitability as instructional tools by veterinarians and students. In addition, the simplified anatomical model was compared to cadaver limbs as instructional tools with regard to the achieved learning success and the self-efficacy of students. The following questions were answered: Does a simulator represent a suitable alternative to a cadaver limb for practicing perineural anesthesia? Does practicing on a simulator increase practical learning success and self-efficacy as much as practicing on a cadaver limb? Does a simulator in the form of a simplified anatomical model and integrated success control provide added value to students? Are the developed simulators suitable for teaching?

## 2 Materials and methods

### 2.1 Ethical declaration

This study was conducted in accordance with the ethical standards of the University of Veterinary Medicine Hannover, Foundation. The study and the questionnaires used were reviewed and approved by the Data Protection Officer of the TiHo prior to implementation. All participants received the data protection notice for the questionnaires and agreed to it with their participation. The handling of personal data was in accordance with the 2016/679 DSGVO. Participation in the study was voluntary. In addition, the study was approved by the thesis committee of the University.

### 2.2 Development of the simulators

Two simulators for practicing perineural anesthesia were developed. The simulators represented the left distal forelimb of a horse, from the hoof to just above the antebrachiocarpal joint. Simulator I (S1) was built as a simplified anatomical model and simulator II (S2) as a more realistic model of the equine distal front limb. An electrically or electronically supported success control was integrated into both simulators. The aim was to perform the following perineural anesthesia on the simulators:

- Modified palmar digital nerve block: *Ramus tori digitalis* anesthesia.

Insertion of needle axial to proximal margin of collateral cartilages, 1–2 cm palmar to palmar border of the deep digital flexor tendon and 2 cm distal advancement along the long axis of the digit, medial, and lateral (21, 22).

- Midpastern palmar digital nerve block (*Nn. digitales palmares*).

Insertion of needle midpastern, abaxial to the deep digital flexor tendon, medial, and lateral (22, 23).

- Modified abaxial sesamoid nerve block (*Nn. palmares*).

Insertion of needle approximately 20 mm above the most distal part of the fetlock joint space, slightly distal to the level of the apex of the proximal sesamoid bones and abaxial to the tendon bundle, medial and lateral (22).

- Palmar metacarpal nerve block (*Nn. metacarpei palmares*).

Axial displacement of suspensory ligament and midmetacarpal insertion of needle axial to splint bone and advancement until contact to palmar aspect of cannon bone (22).

- High four-point nerve block.

Insertion of needles 2–3 cm distal to carpometacarpal joint, subfascially,
(A) lateral (site 1) at the level of the dorsal aspect of the deep digital flexor tendon and medial (site 2) at the level of the dorsal aspect of the superficial digital flexor tendon (*Nn. palmares)* and
(B) axial to the head of the medial (site 3) and lateral (site 4) splint bones and advancement until contact with palmar aspect of cannon bone (*Nn. metacarpei palmares*) (2, 22).

**Figure 1:**
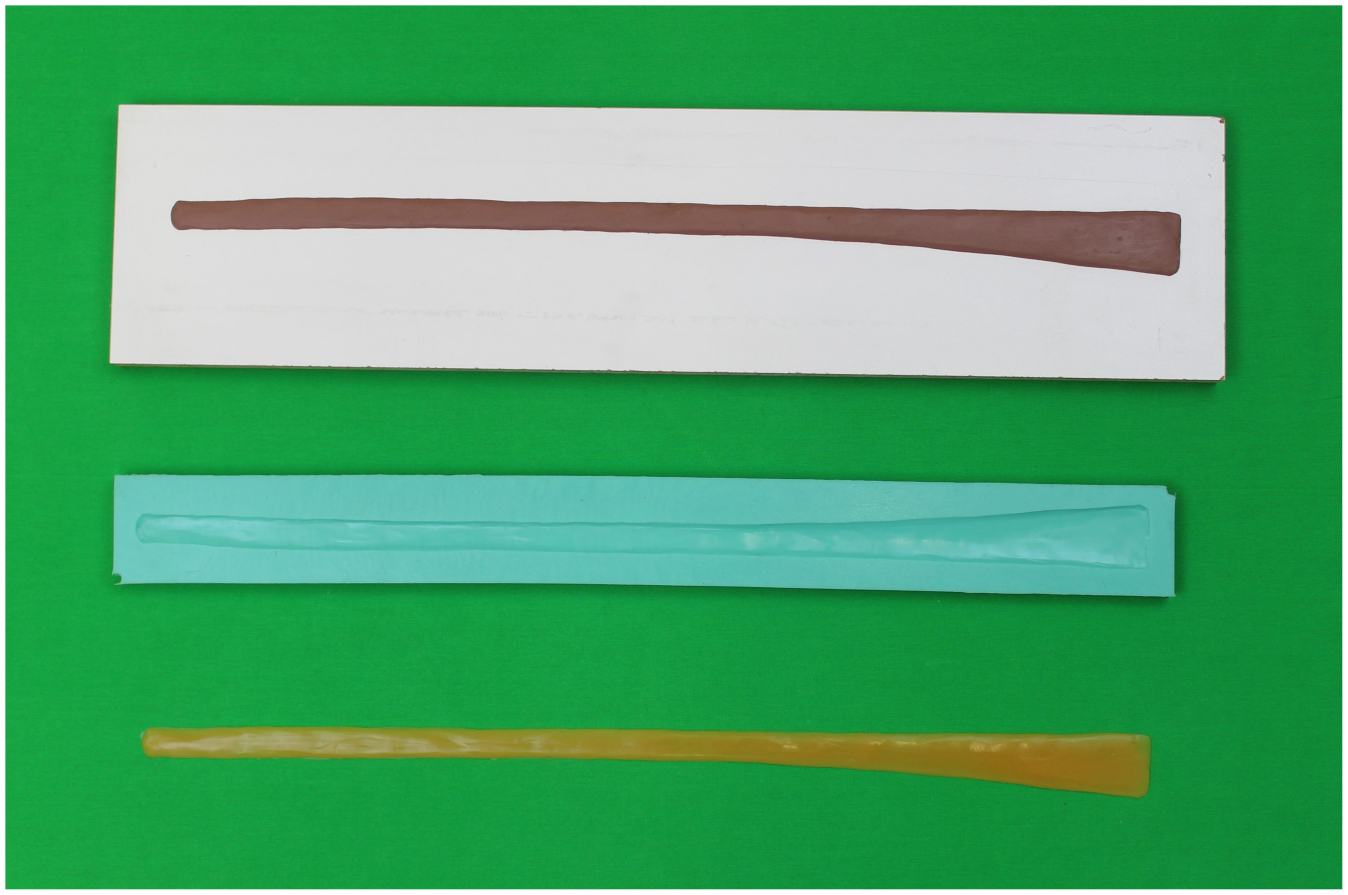
Overview of creating anatomical structures. The model, the negative mold of the common digital extensor tendon and the common digital extensor tendon from silicone are shown.

A commercial bone model made by Synbone AG (Zizers, Switzerland) served as the basic framework in both simulators. The bones of the model were fixed to each other with springs and rubber cords. This allowed flexion of the joints, slightly limited compared to a live horse. Further necessary anatomical structures were modeled with modeling clay, negative molds of which were used for casting anatomical structures (Figure 1). The anatomical structures were cast from materials that were intended to represent the haptics of the structures as realistically as possible. A suspensory ligament, a deep and superficial digital flexor tendon, and a common digital extensor tendon were made from “Ecoflex^®^ 0030” (KauPo Plankenhorn e.K., Spaichingen, Germany), and a digital cushion from silicone “Ecoflex^®^ 0020” (KauPo Plankenhorn e.K.). The ungular cartilages were made from “EpoxAmite^®^” (KauPo Plankenhorn e.K.), which is slightly pliable. Furthermore, a hoof capsule was modeled and then scanned using the 3D-scanner “EinScan Pro 2X 2020” (Shining 3D Tech Co., Ltd., Hangzhou, China) and the Software “EXScan Pro” (Shining 3D Tech Co., Ltd.). After converting the file into a print file with the print software “Preform” (Formlabs GmbH, Berlin, Germany) the hoof capsule was printed from “Black Resin” (Formlabs GmbH) with the SLA printer “Form 3” (Formlabs GmbH).

#### 2.2.1 Simplified anatomical model (S1)

**Figure 2:**
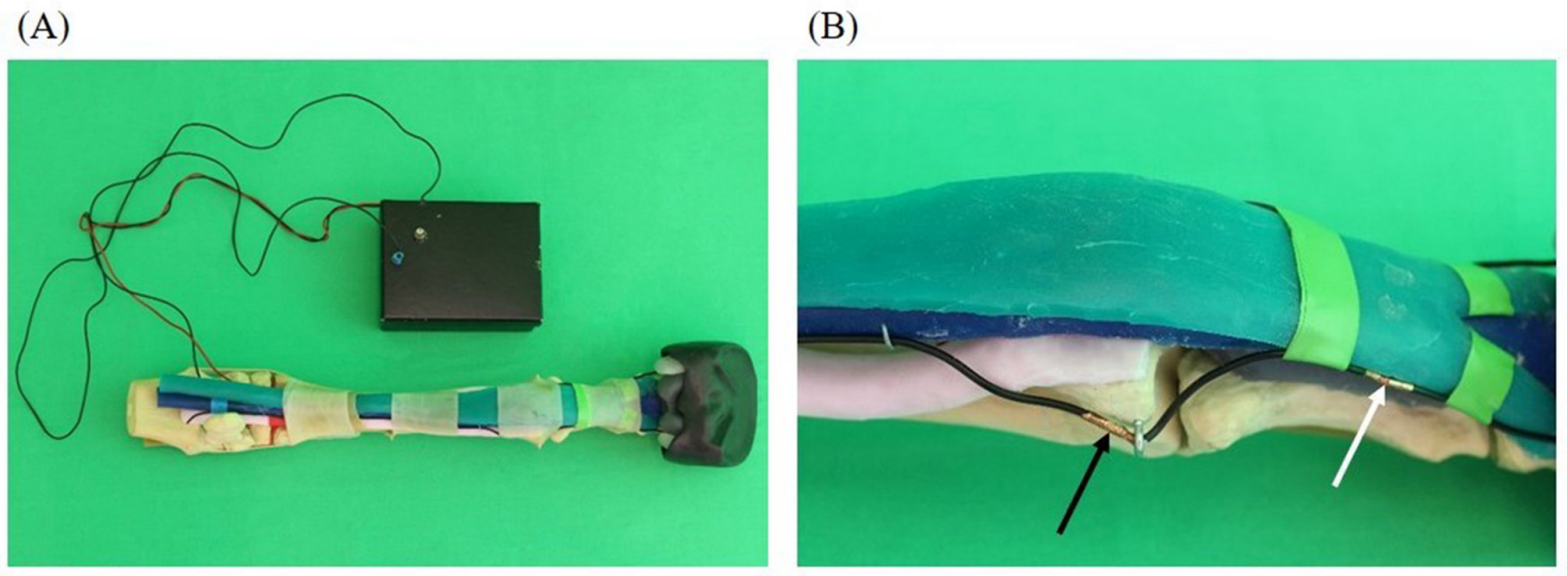
Simplified anatomical model (simulator I): skeleton (beige), superficial digital flexor tendon (green), deep digital flexor tendon (blue), suspensory ligament (light pink), ungular cartilages (white), digital cushion (gray), hoof capsule (black). **(A)** Overview including the integrated feedback system (black box); **(B)** detail showing of the *N. digitalis palmaris lateralis* at the puncture site of the lateral midpastern palmar digital nerve block (white arrow) and the *N. palmaris lateralis* at the puncture site of the Modified abaxial sesamoid block (black arrow).

Simulator I was designed in the form of a simplified anatomical model (Figure 2). The artificial anatomical structures, were fixed to the bone model using glue and screws according to their real positions and courses. Stranded wires were used as nerves (palmar metacarpal nerves, palmar nerves, and palmar digital nerves), which were attached to the other structures using eyebolts and adhesive tape. The stranded wires were stripped at the puncture sites and wrapped with adhesive copper tape. For a more realistic puncture, transparent latex cuffs were made to be placed over the puncture sites for simulating skin. The puncture sites and anatomical structures were palpable and visible on this simulator.

An electric circuit was built into the simulator to integrate a tool for immediate success control. The circuit consisted of a battery that was stored in a cardboard box. The circuit integrated the stranded wires that served as nerves, a needle connected with a cable and a light, which was located in the lid of the cardboard box. If the integrated needle hit one of the prepared puncture sites during a puncture, the lamp lighted up.

Simulator I just allowed puncture with an integrated needle, but did not allow the application of fluid. The aim was to give students the opportunity to gain a visual and haptic impression of the anatomical conditions and, above all, the most important anatomical landmarks before and during the puncture.

#### 2.2.2 Realistic model (S2)

**Figure 3:**
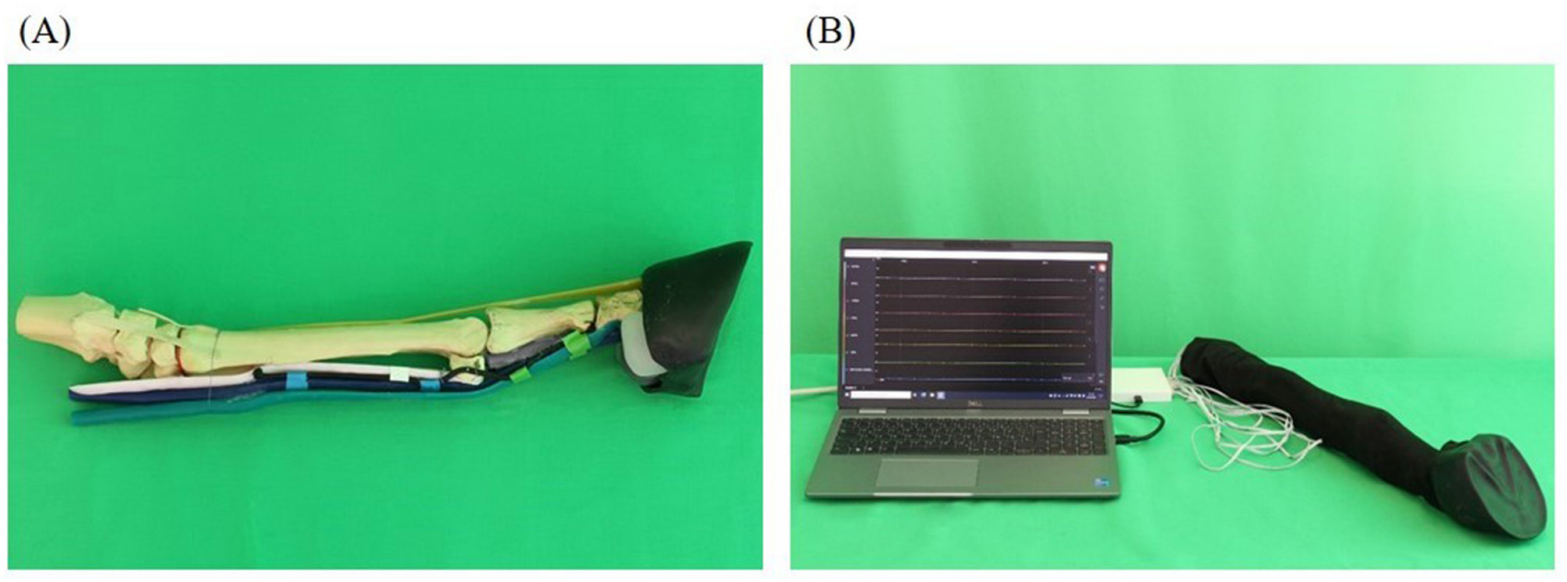
Realistic model (simulator II): skeleton (beige), superficial deep tendon (green), deep flexor tendon (blue), suspensory ligament (light pink), common digital extensor tendon (orange), ungular cartilages (white), digital cushion (gray), hoof capsule (black). **(A)** Basic framework without prepared puncture sites and fleece cover; **(B)** complete model covered by fleece as skin substitute with integrated feedback system.

As S2 a more realistic model compared to S1 was created (Figure 3). This included that the fabricated anatomical structures were attached to the bone basis. The nerves were simulated with rubber cords. A fleece cover was pulled over the model to simulate the skin. The cover could be opened with a zipper and easily removed.

A success control was integrated into the simulator which was based on the principle of conductivity measurement. At the level of the puncture sites, latex cuffs were placed over the anatomical model into which pieces of an electrically conductive fabric (Holland Shielding Systems BV, Dordrecht, Netherlands) were glued. The pieces of fabric were previously connected with a cable. Additional pieces of fabric equipped with cables were also placed on the inside of the fleece cover at the level of the puncture sites and fixed to the fleece cover by gluing pieces of latex over them. The pieces of fabric served as electrodes and were connected to a circuit board in a plastic housing via cables. The circuit board was connected to the measuring device “Saleae Logic Pro 16” (Saleae, South San Francisco, USA). The measuring device was connected to a laptop, model “Dell Latitude 5520” (Dell GmbH, Frankfurt am Main, Germany), where the Logic Analyzer software “Logic 2.4.1” (Saleae) was used. The program showed 11 graphs arranged one below the other. Every graph was assigned to one puncture site on the simulator and named accordingly. The x-axis showed the time and the y-axis showed the voltage measured at the puncture sites. When a puncture site was touched with a needle, the voltage increased leading to an increase of the corresponding curve as long as the needle was kept in contact with the simulator.

The electronic design of the integrated success control was carried out in cooperation with the company JSlabs GmbH (Wunstorf, Germany).

On S2 perineural anesthesia could be performed in the same way as on a live horse. In addition to puncture using a freely selectable needle, it was also possible to apply fluid.

### 2.3 Intervention study with students in their clinical year

**Figure 4:**
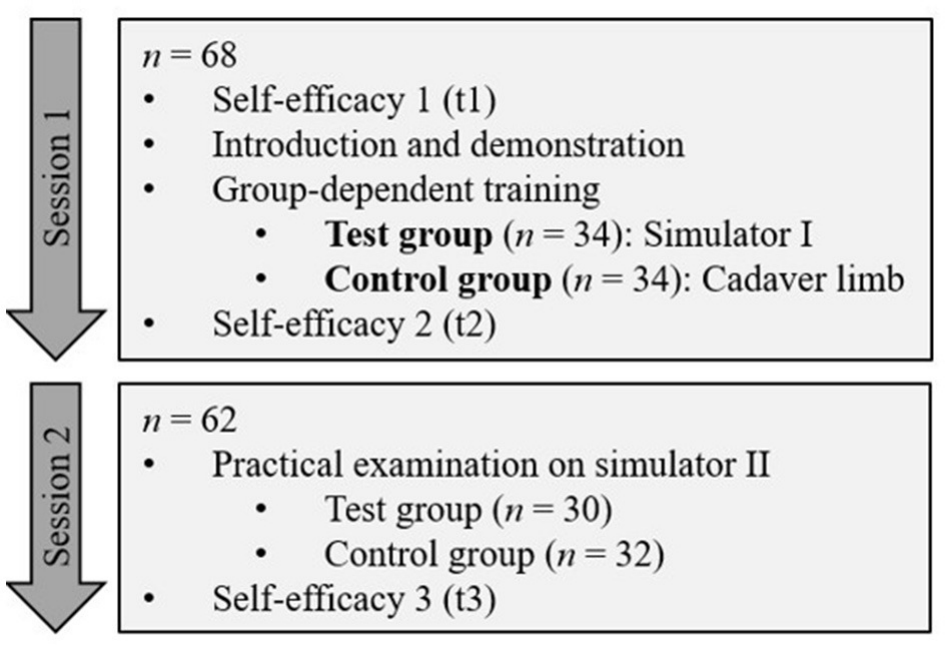
Process of the intervention study with students in their clinical year (*n* = 68/62).

To test the suitability of S1 as an instructional tool an intervention study was conducted. The study population were 5^th^-year students completing their 10-week clinical rotation as part of their clinical year at the Clinic for Horses of the TiHo in 2022/2023. The study was integrated into the regular practical perineural anesthesia exercise during the rotation and the study population was divided into four groups (cycles), which completed the rotation spread over the year. Figure 4 shows the process of the study.

#### 2.3.1 Use of S1 for training

Simulator I was used as an instructional tool in the study and compared with the exercise on the cadaver limb.

A total of 68 students participated in session 1. The session took place in groups of 15–20 students. At the beginning, students received a 20–30 min introduction to diagnostic perineural anesthesia by the same instructor. The puncture sites and techniques were demonstrated in practice on a cadaver limb.

After the demonstration, participants were divided into two groups of approximately 7–10 students using a random number generator (24).

The test group received supervised training on S1. Two simulators I were available for one group so that small groups of 3–5 students were created who practiced together on one simulator. After a short introduction to the use and functions, the students were first able to repeat the anatomical structures of the limb with the help of the simulators. They were then allowed to perform punctures independently on the simulator and repeat them several times. During this process, they received supervision from a member of the CSL staff.

The control group practiced punctures on cadaver limbs. The students punctured the puncture sites and injected ink. A small group of 2–3 students worked together on one cadaver limb so that each student could repeat the placement of needles as desired and finally inject a maximum of one dose of dye at each injection site. They then dissected the cadaver limbs to verify that they had injected the ink at the correct sites. In doing so, they also had the opportunity to repeat these anatomical structures. The group was supervised by two clinicians from the Clinic for Horses of the TiHo.

Session 1 lasted 90 min in total.

#### 2.3.2 Practical examination

In order to be able to review the practical learning success of the students, session 2 with an examination took place approximately 7–10 days after session 1, in which 62 students participated.

The examination took place in the form of an Objective Structured Clinical Examination (OSCE) station format which was developed by the TiHo (25). Students were supposed to perform a Modified abaxial sesamoid nerve block and Palmar metacarpal nerve block independently and without assistance within a given time frame of 14 min. The S2 served as the simulation patient. The integrated success control was disabled during the assessment in order not to influence students' performance.

Students' performance was assessed using a checklist (Supplementary Document 1) and was evaluated by an independent CSL staff member. The checklist contained 53 items describing the individual steps to be performed. Items could be scored as “fulfilled” and “not fulfilled” and two items could additionally be scored as “partially fulfilled.” The individual items were rated in advance by allotting 1–4 points.

The checklist was developed in cooperation with the Clinic for Horses of the TiHo based on specialist literature and formally reviewed by the E-Learning Consulting of the TiHo. Each checklist was marked with an individual code for assignment.

#### 2.3.3 Investigation of experience and self-efficacy

At three different points in time within the study, participants received a questionnaire about experiences and self-efficacy (Supplementary Document 2).

The first questionnaire was used to determine how often students had performed perineural anesthesia on a cadaver, a simulator, and a live animal prior to study participation. The subsequent self-efficacy questions were divided into two blocks: theoretical knowledge and practical skills. The students assessed their theoretical knowledge of equine limb anatomy, puncture sites, and performance of diagnostic perineural anesthesia. Furthermore, they assessed their practical skills regarding the performance of perineural anesthesia, both in finding the correct puncture sites and their anesthetic effect. Each block of questions on self-efficacy contained six items that could be assessed using a five-point Likert scale (“Does not apply” = 1, “Somewhat does not apply” = 2, “Partially applies” = 3, “Somewhat applies” = 4, and “Fully applies” = 5).

The participants completed the questionnaires at the beginning of session 1 (t1), at the end of session 1 (t2), and at the end of session 2 (t3). The questionnaires were marked with an individual code for assignment.

The questionnaires were checked in advance by CSL staff for comprehensibility and then formally reviewed by the E-Learning Consulting of the TiHo.

### 2.4 Evaluation of the simulators

The two simulators were evaluated using questionnaires to assess their realism and suitability as instructional tools.

The questionnaires for the two evaluation groups were similarly designed.

First, the respondents were asked what difficulties they perceived while performing perineural anesthesia.

The simulators could be evaluated for realism and suitability based on 10–11 items using response options on a four-point Likert scale (“Does not apply” = 1, “Somewhat does not apply” = 2, “Somewhat applies” = 3, “Fully applies” = 4, “No answer”). For each simulator, there was also the possibility to name optimization needs/improvement suggestions and comments/ideas within free text fields. Furthermore, questions were asked about the use of the simulators and the favored instructional tool could be named.

#### 2.4.1 Evaluation by students

Following the intervention study, another training session took place for the participants in a cross-over design. The training session was similar to session 1 of the study apart from a change of groups. The former study group received the practical training on cadaver limbs and the control group on S1. Thus, all students had the opportunity to perform the exercise on cadaver limbs usually used as instructional tools and additionally on S1 as part of the study.

Subsequently, the 5^th^-year students were able to evaluate the applied simulators using a questionnaire. Forty-nine students participated in the evaluation.

#### 2.4.2 Evaluation by experts

The simulators were also evaluated by 7 veterinarians from the Department of Surgery and Orthopedics at the Clinic for Horses of the TiHo. The veterinarians were introduced to the operation and functionalities of the simulators by a CSL staff member. Afterwards, they had the opportunity to touch the simulators and try them out independently. They also evaluated the simulators using a questionnaire.

### 2.5 Statistical analyses

Data analysis was performed using Microsoft^®^ Office Excel 2016 (Microsoft Corporation, Redmond, WA, USA) und SAS^®^ Software, Version 9.4 and SAS^®^ Enterprise Guide^®^ 7.1 (SAS Institute Inc., Cary, NC, USA). *P*-values below 0.05 were assumed to be significant.

The Pearson's chi-squared test was used to investigate whether the groups differed in terms of their experience before the study. Students were considered experienced if they had already performed perineural anesthesia on live horses, cadaver limbs, or simulators prior to the study.

The results of the practical examination were analyzed with descriptive statistics. The points of the individual items were added up and a total score was calculated for each student. In addition, the number of correct puncture sites was counted for each student. Therefore, three individual items were considered, which queried the identification of correct puncture sites:

- Identification of the correct puncture site.
- Palpation of the neurovascular bundle and the medial palmar nerve approximately 20 mm proximal to the most distal part of the fetlock joint space, near the apex of the medial proximal sesamoid bone, abaxial to the superficial and deep digital flexor tendon.
- Identification of the correct puncture site.
- Palpation of the neurovascular bundle and the lateral palmar nerve approximately 20 mm proximal to the most distal part of the fetlock joint space, near the apex of the lateral proximal sesamoid bone, abaxial to the superficial and deep digital flexor tendon.
- Identification of the correct puncture site.
- Lateral approximately 3–4 cm proximal to the distal end of the lateral splint bone or halfway along the metacarpus, between the suspensory ligament and lateral splint bone.

Using a full factorial linear model, the influence of experience, cycle, group, and the interactions between these factors on the achieved total score was investigated. The factors experience and cycle were regarded as potential nuisance parameters.

The self-efficacy score was calculated by adding up the points given to the individual items. A score was calculated for each student and each point in time. The results were analyzed with descriptive statistics. A linear mixed model was used to evaluate the influence of cycle, experience, group, and all interactions between these factors as well as the point in time and its interaction with group on the self-efficacy score. Also, here the factors experience and cycle were regarded as potential nuisance parameters and the correlation structure was included as above as an autoregressive [AR(1)] model over time.

The items “I have extensive theoretical knowledge regarding the anatomy of an equine limb.” and “I know the course and location of the relevant nerves of an equine limb for performing perineural anesthesia.” were considered individually, described using descriptive statistics. A sign- test was used to test the increase for both items and both groups individually for significance and compared between groups using Pearson's chi-squared test.

Evaluation results were analyzed descriptively and are presented graphically. Diverging bar charts are used to show results of Likert-type questions. Free text responses were analyzed qualitatively.

## 3 Results

### 3.1 Intervention study

The groups did not differ in terms of their experience at t1 (*p* = 0.38).

Sixty-two students participated in the practical examination. A total score of 107 points could be achieved. The lowest score was 35.98 % (38.5 points) and the highest score was 92.52% (99 points).

**Figure 5:**
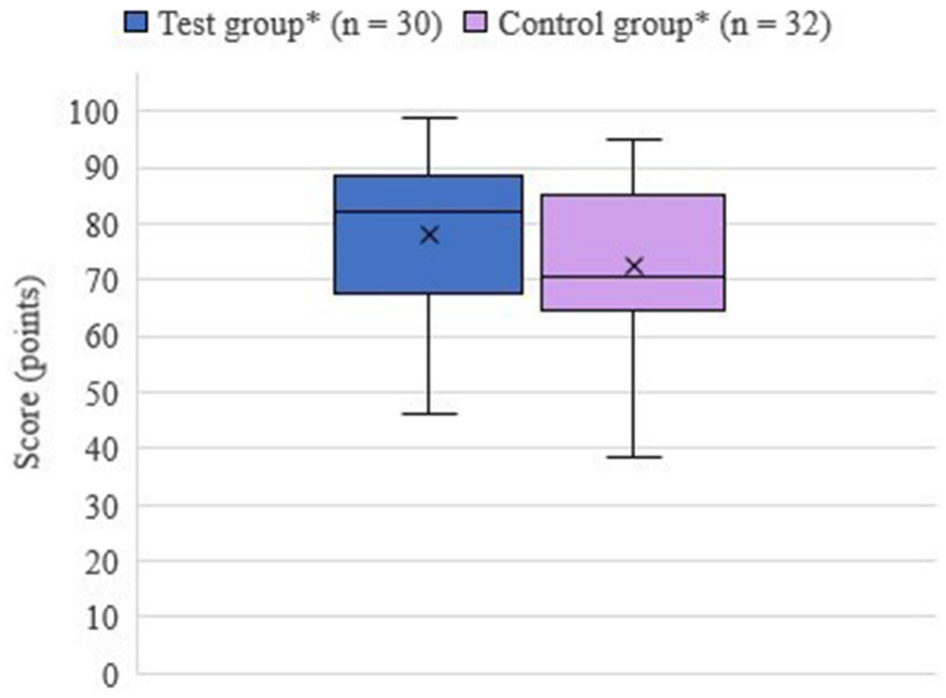
Achieved total score in practical examination on simulator II depending on group; *test group: training on simulator I, control group: training on cadaver limbs; x, mean value.

The results of the practical examination are shown in Figure 5. The test group achieved a mean score of 72.9% (78 points) and the control group achieved a mean score of 67.76% (72.5 points). The test group correctly located an average of 2.2/3 puncture sites, the control group 2/3 puncture sites. Despite the mean difference of 5.14% in the number of points achieved and 0.2 in the number of correct puncture sites, no significant difference was found between the test group with training on the S1 and the control group with training on cadaver limbs. The group had no significant effect on the achieved score (*p* = 0.36). There were also no effects of the other factors or interactions investigated.

The first questionnaire for self-efficacy at t1 was completed by 67 participants. The second questionnaire at t2 was completed by 68 participants and the third questionnaire at t3 by 62 participants. If a participant did not clearly evaluate all items in a questionnaire, no self-efficacy score was calculated for this participant at that time and was thus not included in the descriptive statistics.

**Figure 6:**
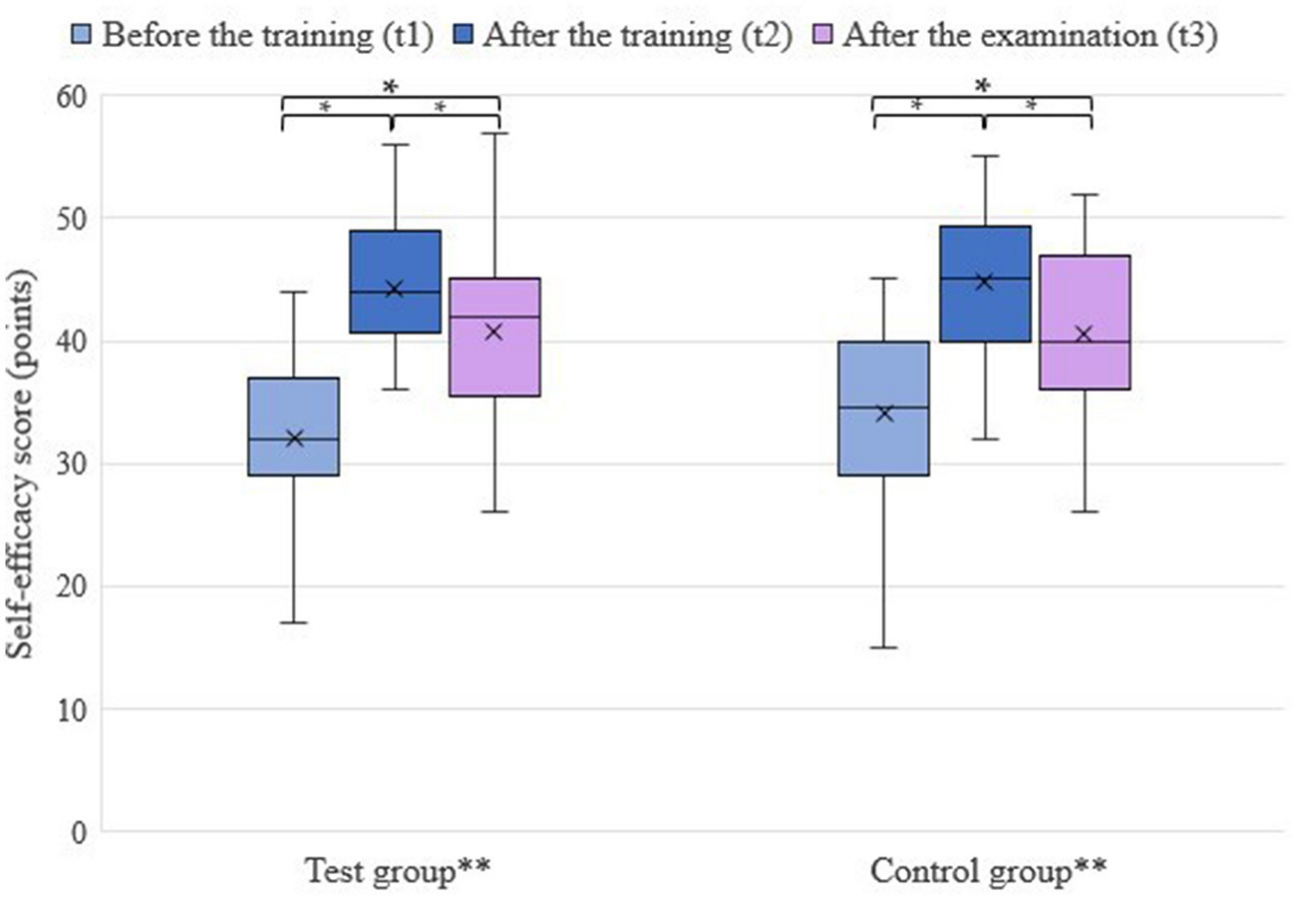
Self-efficacy score depending on group and point in time; *p-value below 0.05; **test group: training on simulator I, control group: training on cadaver limbs; x, mean value.

For self-efficacy, total scores between 15 and 57 per student were achieved. In both groups, an increase in self-efficacy was observed at t2. The test group had a mean self-efficacy score of 32.1 (*n* = 31) at t1 and 44.24 (*n* = 34) at t2. In comparison, the control group had a mean self-efficacy score of 34.09 (*n* = 34) at t1 and 44.85 (*n* = 34) at t2. At t3, the self-efficacy score dropped in both groups. The test group reached a score of 40.75 (*n* = 28), and the control group 40.63 (*n* = 32). The results are shown in Figure 6.

The group assignment for training had no significant influence on the self-efficacy score (*p* = 0.80) and the self-efficacy score of the groups did not differ significantly at any point in time (*p* = 0.60). Experience had a positive effect on self-efficacy score (*p* = 0.020) and there was an interaction between the factor cycle, experience, and group (*p* = 0.039).

The results of the analysis of selected items are shown in Table 1. The self-efficacy scores for the selected items regarding anatomical knowledge did not differ significantly between the groups at any point in time. In both groups, the self-efficacy scores for both items increased after the training. For the second item “I know the course and location of the relevant nerves of an equine limb for performing perineural anesthesia.” the scores increased significantly in both groups.

**Table 1:** Self-efficacy regarding anatomical knowledge in the two groups (*Md, SD*).

	**Test group** ^*^		**Control group** ^*^	
	**t1** ^**^	**t2** ^**^	**t1** ^**^	**t2** ^**^
I have extensive theoretical knowledge regarding the anatomy of an equine limb.	3 ± 0.67 (*n* = 33)	4 ± 0.56 (*n* = 34)	4 ± 0.56 (*n* = 34)	4 ± 0.53 (*n* = 34)
I know the course and location of the relevant nerves of an equine limb for performing perineural anesthesia.	3^a^ ± 0.65 (*n* = 33)	4^a^ ± 0.61 (*n* = 34)	3^b^ ± 0.77 (*n* = 34)	4^b^ ± 0.48 (*n* = 34)

^*^Test group: training on simulator I, control group: training on cadaver limbs; ^**^t1: before the training, t2: after the training; ^a, b^values with the same key figure differ significantly, p-value below 0.05 (response options “Does not apply” = 1, “Somewhat does not apply” = 2, “Partially applies” = 3, “Somewhat applies” = 4, “Fully applies” = 5).

### 3.2 Evaluation

The questionnaires for the evaluation were completed by 49 students who had participated in the intervention study. In addition, 7 staff veterinarians with different experience levels from the Department of Surgery and Orthopedics of the Clinic for Horses of the TiHo participated in the evaluation survey.

Upon the first question about the general difficulties while performing perineural anesthesia, the students stated that finding the anatomical structures and the correct puncture site as well as the correct placement of the needle, including the puncture depth, were the main difficulties they experienced when performing. They also mentioned the horse's movements or cooperation as a potential challenge. As well as the avoidance of puncture errors, anatomical variability and lack of practice or opportunity were also perceived as difficulties.

The veterinarians often stated that they considered movement or non-cooperation of the horse during blocking a difficulty. In addition to the complexity of anatomical structures and their variance, the different ways of performing perineural anesthesia depending on the examiner were also perceived as a difficulty.

#### 3.2.1 Evaluation of S1 (simplified anatomical model)

**Figure 7:**
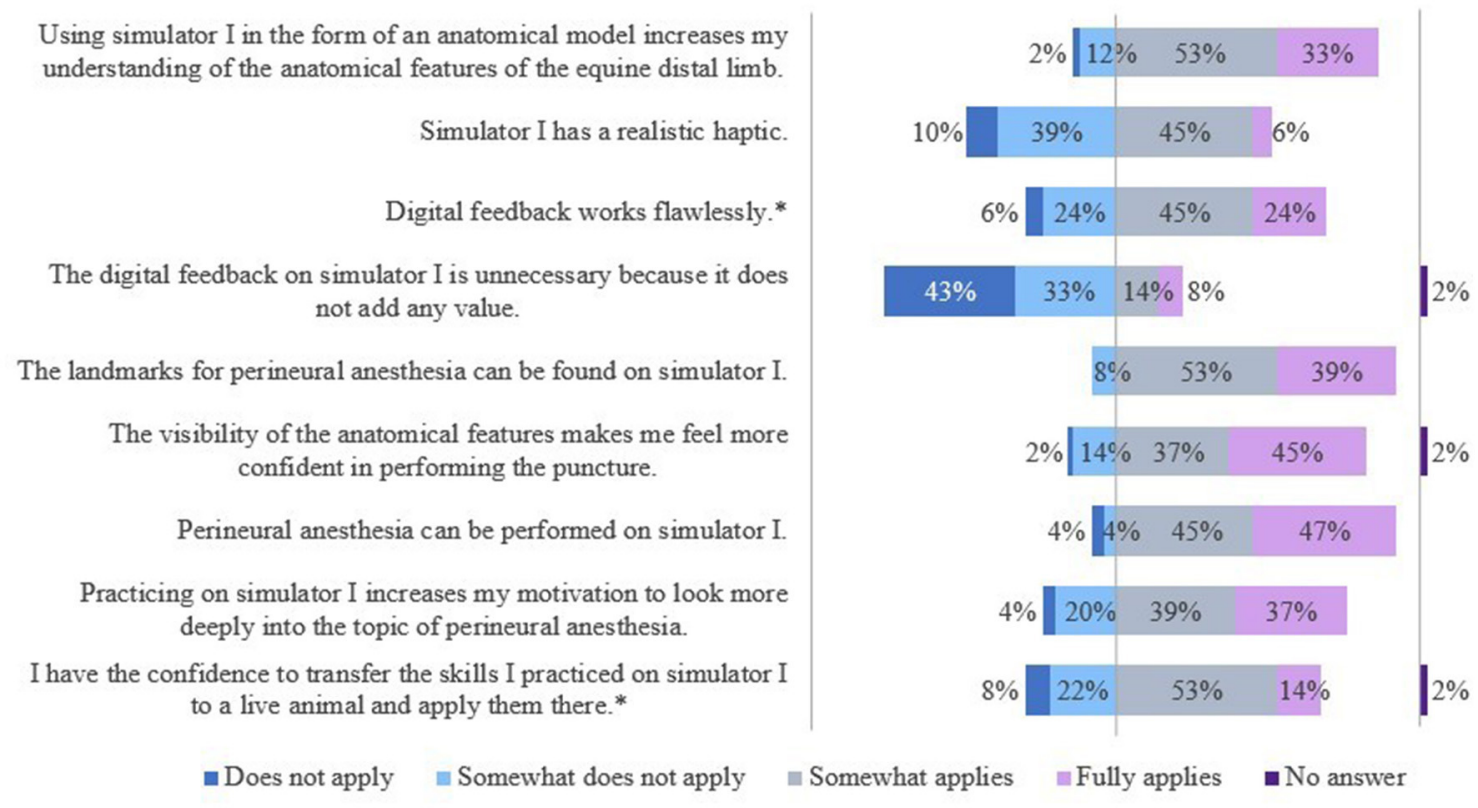
Evaluation of simulator I by students of the 5^th^ year. *Due to rounding, the total is not exactly 100%.

**Figure 8:**
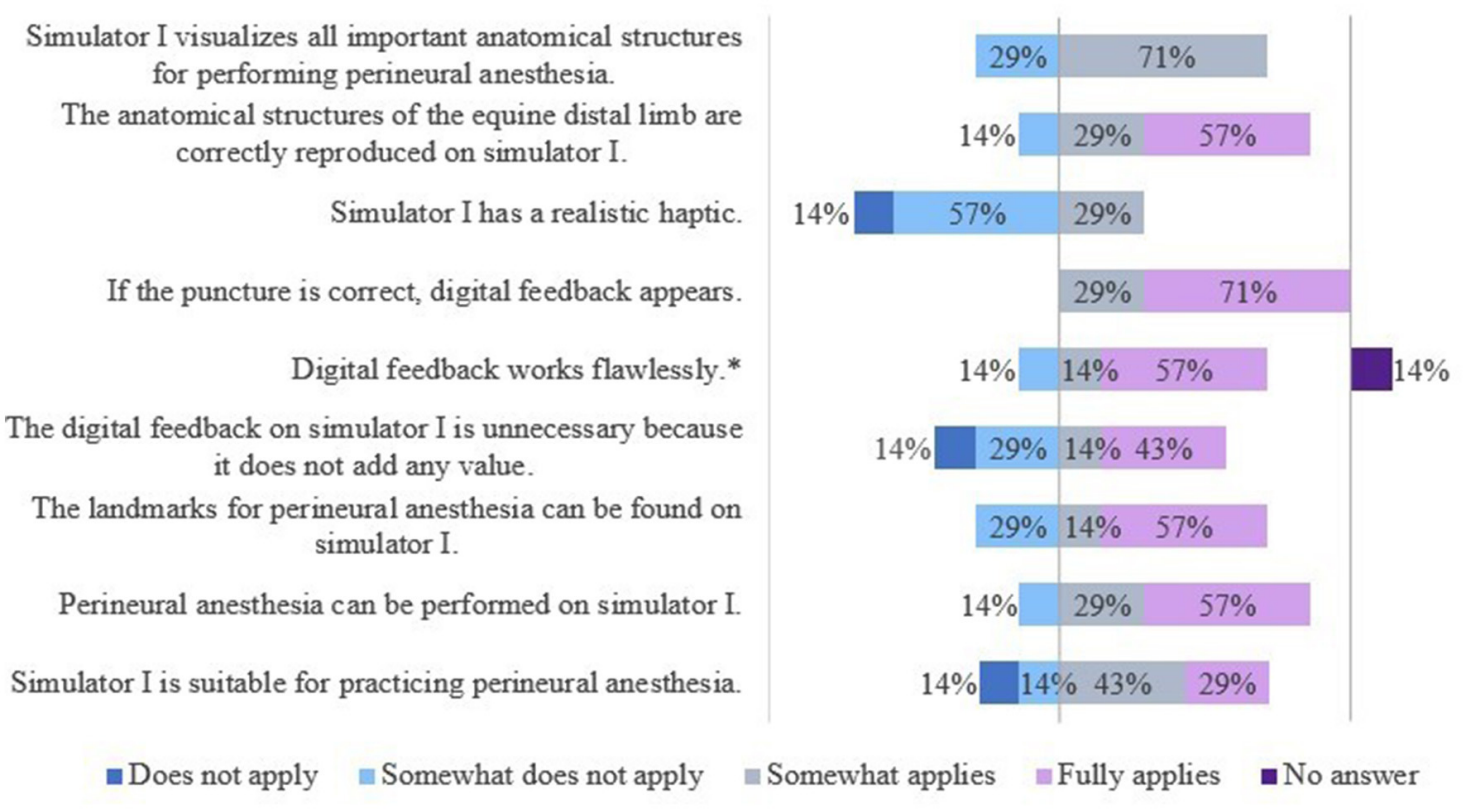
Evaluation of simulator I by veterinarians. *Due to rounding, the total is not exactly 100%.

The results of the evaluation of the simulators' features are shown in Figures 7, 8.

The students saw a need to optimize the haptic of the splint bone. They also criticized the tactility of the “nerve tract,” especially in the fetlock region. Some students criticized the visibility of the puncture sites and suggested that an opaque skin-like cover would be a valid alternative. Furthermore, it was noted that the puncture depth of the needle was not relevant and diffusion of the local anesthetic or dye was not taken into account, but that the “nerve” had to be hit exactly.

The veterinarians saw a need to optimize S1 mainly with regard to its haptics and the extent of the displayed anatomical structures. Additional anatomical, especially synovial structures were desired.

#### 3.2.2 Evaluation of S2 (realistic model)

**Figure 9:**
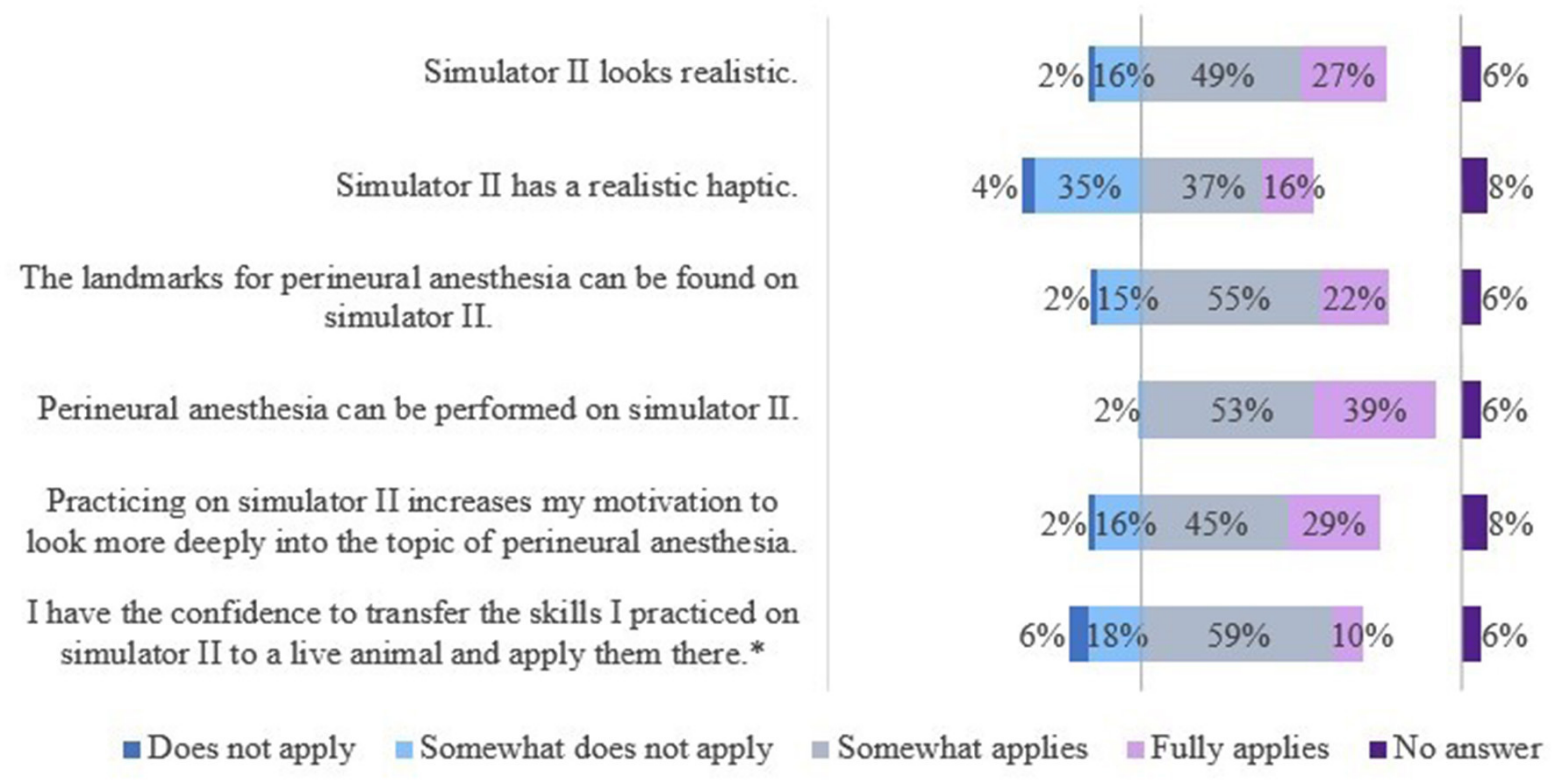
Evaluation of simulator II by students of the 5^th^ year. *Due to rounding, the total is not exactly 100%.

**Figure 10:**
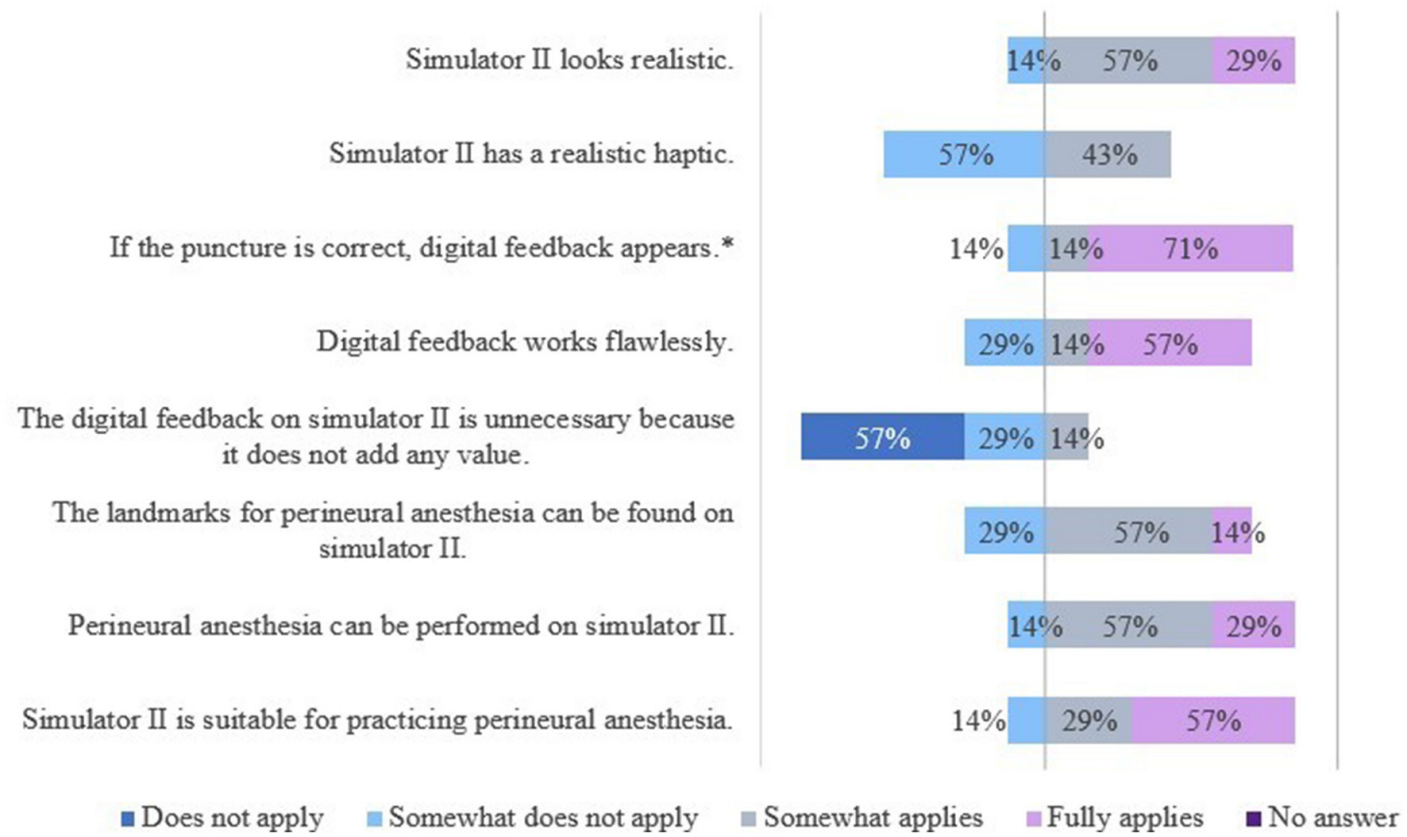
Evaluation of simulator II by veterinarians. *Due to rounding, the total is not exactly 100%.

The results of the evaluation of the simulators' features are shown in Figures 9, 10.

For S2 students saw a need to optimize the haptic of the splint bones, analogous to S1. The veterinarians wished to optimize the skin-like cover. A thicker cover would have been more realistic and preferred. Students and veterinarians mentioned the poor tactility of the “nerve tract” in some locations, especially in the fetlock region.

#### 3.2.3 Application of the simulators

All student respondents and all 7 veterinarians agreed that practicing perineural anesthesia on cadaver limbs or simulators was useful before performing it on live animals. All students and 6 of the veterinarian respondents were of the opinion that more practical training would be worthwhile.

When deciding on a preferred instructional tool, the majority of the students (77.55%) chose the cadaver limb. Only two students (4.08%) opted for the S2 and one student (2.04%) for the live animal. All veterinarians selected the cadaver limb. The S1 was not preferred by anyone. Multiple responses (16.33%) were not evaluated.

The survey finally asked about general aspects of training tools and their importance. The students considered realism ($\tilde{x}$ = 4 ± 0.37) and feedback/verifiability of performance ($\tilde{x}$ = 4 ± 0.61) most important, followed by animal welfare ($\tilde{x}$ = 3 ± 0.91), sustainability ($\tilde{x}$ = 3 ± 0.87), ease of use ($\tilde{x}$ = 3 ± 0.87), and availability ($\tilde{x}$ = 3 ± 1.05). The veterinarians also indicated that realism ($\tilde{x}$ = 4 ± 0.38) and feedback/verifiability of performance ($\tilde{x}$ = 4 ± 0.49) were most important to them in an instructional tool, ahead of sustainability ($\tilde{x}$ = 3 ± 0.69), animal welfare ($\tilde{x}$ = 3 ± 1.41), ease of use ($\tilde{x}$ = 3 ± 1.41), and availability ($\tilde{x}$ = 3 ± 0.41).

## 4 Discussion

In the present study, two simulators for teaching the clinical performance of equine perineural anesthesia with integrated success control were developed, built, and subsequently evaluated by students and veterinarians. Simulator I was designed as a simplified anatomical model and compared to cadaver limbs for its suitability as an instructional tool. The more realistic S2 was used as a simulation object for verification of the practical learning success.

The study provides evidence that the developed simulators are suitable as instructional tools to precede and complement cadaver exercises for teaching students how to perform perineural anesthesia, even with limitations in terms of realism. Their integrated success control enables immediate feedback concerning correct puncture and numerous repetitions of the procedure. An exercise on S1 led to similar practical skills and an increase in self-efficacy among students equivalent to the same exercise on the cadaver limb.

The need of using simulators for teaching equine perineural anesthesia arose from the fact that exercises on cadaver limbs are associated with a high logistical and hygienic effort (9). In addition, due to the limited time available in the 2^nd^ and 3^rd^ years of study, exercises with cadaver limbs are currently not offered to all TiHo students, but only to those on rotation at the Clinic for Horses. Simulators have the logistical and hygienic advantage (9) that they can also be used in a clean environment outside of wet labs, e.g., the CSL. In general, ethical concerns should also be taken into account when using cadavers for teaching purposes (9). Since the cadaver limbs used at the Clinic for Horses originate from ethically sound sources (26) and students are trained to handle cadavers appropriately, this aspect can be largely neglected.

Extensive knowledge of equine limb anatomy is essential in order to perform and interpret perineural anesthesia correctly (5, 27). The evaluation results collected in this study show that the complex anatomy and identification of anatomical landmarks on the horse's limbs are considered a challenge when performing perineural anesthesia. The students' anatomical understanding increased as a result of practicing on the S1. The self-efficacy survey also revealed a subjectively perceived increase in anatomical knowledge, particularly with regard to the course and location of the relevant nerves of an equine limb for performing perineural anesthesia. An equivalent increase was also observed among the students who practiced on the cadaver limb. The literature recommends practicing the perineural anesthesia on cadaver limbs and then dissecting them in order to control the punctures on the one hand, and to repeat the anatomical conditions on the other hand (5). Consistent with the results obtained, Gunning et al. (8) found no significant difference when they compared a simulator with a cadaver limb as an instructional tool for perineural anesthesia. However, a study about training methods for infiltration techniques in human medicine found that practicing on cadavers produced better OSCE results than practicing on anatomical models (28). Further studies came to different conclusions when comparing cadavers and simulators as instructional tools for surgery training (29–31). The current study shows that in addition to the recommended instructional tools in the literature, the developed anatomical model is also suitable for practicing perineural anesthesia and repetition of the most relevant anatomical landmarks. At the same time, cadaver limbs allow the learner to refresh knowledge on a plethora of further anatomical structures.

Another difficulty when performing nerve blocks is evaluating the correctness or potential efficacy puncture. On a live horse, the skin sensitivity in the anesthetized area can be used as an indication. Otherwise, it can only be assumed with certainty that a nerve has been infiltrated if the nerve block is positive (3). On cadaver limbs, the puncture can be verified by injecting dye and then dissecting the limb (5). The diffusion behavior and distribution of the fluid are simulated and inadvertent intrasynovial injections can be identified. However, this is possible only once per site on a cadaver limb. As with Gunning et al. (8), immediate success controls were integrated into the developed simulators. On S1, the need for feedback was viewed critically by the experts, which may be explained by the fact that a visual control of the puncture is possible on the simulator even without a feedback mechanism. The majority of experts were of the opinion that the feedback mechanism on S2 offers a benefit. The verifiability of implementation and feedback were important to both evaluation groups. In a previous study, Mahmood and Darzi (17) found that repeated practice on a colonoscopy simulator did not result in any improvement in skills if the learners did not receive feedback. This shows that feedback as a didactic method is also essential for simulation-based training. Immediate feedback is preferred by learners and leads to greater engagement from learners (32, 33). According to Al Fayyadh et al. (32) it is easier to perceive auditory feedback when the performance is visual, as it is largely the case with the S1. In summary, it can be said that the possibility to verify the success of the punctures is important and necessary. A feedback mechanism in instructional tools is positive for learning success, whereby immediate feedback is even more beneficial. In addition to visual feedback, auditory feedback in the form of a buzzer will also be used on S1 in the future. Furthermore, a feature could be added that also recognizes malpositioning of the needle, e.g., inadvertent centesis of synovial structures. The use of liquids such as water or dye solutions to simulate an injection is challenging on a perineural anesthesia simulator because, among other things, an absorbent material must be used as subcutaneous tissue, the simulator must be dried and contamination from dyes must be removed. Simulator II allowed the application of a small volume of water, but its distribution was non considered in the feedback.

The quality of the developed simulators was evaluated by veterinarians and 5^th^-year students in terms of “face validity” and “content validity” (34). The evaluation results showed deficiencies in the haptics of both simulators (face validity). For didactic and technical reasons, the simulators were designed to reproduce the anatomical conditions of the equine limb in a very simplified way, which limits realism of the haptics. The haptics are also affected by the technically necessary attachment of the anatomical structures. During the development of the simulators, great attention was given to ensure that the landmarks for locating the puncture sites were present (content validity), which is the case when looking at the evaluation results. The realism was rated as the most important criterion of an instructional tool, so both evaluation groups largely preferred the cadaver limb for practicing perineural anesthesia. Simulators can be categorized according to their realism into high-fidelity, mid-fidelity, and low-fidelity (10, 35). The cadaver limb can be seen as a high-fidelity instructional tool (9), whereas S2 is a mid-fidelity simulator, which allows the procedure to be performed in a realistic way. Simulator I is more a low-fidelity simulator. In other studies comparing different instructional tools, respondents also preferred the more realistic training objects (11, 36, 37). It was also shown that the learning success does not necessarily depend on the realism of the training object (38–41). The results of our study are thus consistent with the literature.

In the present study, self-efficacy was assessed once at the beginning, after the training session with the different instructional tools, and after the practical examination. Self-efficacy was described by Bandura (42) and describes the self-assessment of being able to cope with a difficult task or situation using one's own skills and knowledge (42). A high self-efficacy gives one confidence and therefore has a positive impact on performance on a live animal (16). Klassen and Klassen (43) recommend that self-efficacy values be collected at least three times during a study in order to achieve evidence-based comparability. With regard to the comparability of the instructional tools, the students' self-efficacy at the time after the training session was of particular interest. The group assignment had no significant influence on the self-efficacy score, but the self-efficacy scores of both groups increased significantly after the training sessions. It can be concluded that practicing on S1 as a simplified anatomical model increases self-efficacy, as does practicing on a cadaver. This is completely in line with existing literature. Several studies have shown that simulation-based training can increase self-efficacy (6, 7, 11–13). Giese et al. (7) investigated the influence of different instructional tools for bovine rectal palpation on self-efficacy and found out that high- and mid-fidelity simulators increased self-efficacy to the same extent. Similarly, Michels and Vanhomwegen (28) who investigated different instructional tools for infiltration techniques found significant increases in self-efficacy after the exercises, but no significant differences between the training strategies. Practical exercises on instructional tools can generally increase self-efficacy and this does not depend on their realism.

The strength of the study was the development and evaluation of two simulators with different fidelity and the additional comparison to the usual instructional tool cadaver limb. By integrating the intervention study into the regular practical exercise for perineural anesthesia during the clinical year, a comparison between the simulator and a cadaver limb in the current standard exercise was possible. In session 1, the students all received the same introduction and demonstration only with a cadaver forelimb in order to avoid bias. However, this has to be considered as a factor potentially having a positive influence on the relevant anatomical knowledge of the students in both groups, as anatomical landmarks were demonstrated in detail during preparation of the cadaver limb.

A minor limitation of the prototype simulators is that they only reflect forelimb anatomy, while using cadaver limbs usually an equivalent number of fore- and hindlimbs can be provided. This aspect has to be taken into account when teaching nerve blocks that imply consideration of neuroanatomical variations between fore- and hindlimbs (e.g., relevance of deep branch of lateral plantar nerve, dorsal metatarsal nerves in hindlimbs).

In addition, the simulators currently do not allow a conventional low four-point nerve block to be performed as described in the Anglo-American literature (1–3). Blocking the palmar nerves distally can only be simulated in the form of a modified abaxial sesamoid nerve block, while blocking the palmar metacarpal nerves is simulated with a puncture technique in the mid-metacarpal region and palmar to the splint bones (2). This means that the puncture sites are more distal for the palmar nerves and more proximal for the palmar metacarpal nerves compared to a conventional low four-point block. However, this combination of a modified abaxial sesamoid nerve block and (a) an alternative palmar metacarpal nerve block (simulator) has an effect comparable to a conventional low four-point nerve block in a clinical setting. At the TiHo Equine Hospital, another alternative palmar metacarpal nerve block technique (b) is performed using a different method to that described for the simulator: The needle is inserted 1–2 finger widths proximal to the distal aspect of the splint bone between the splint bone and the cannon bone until the interosseous metacarpal ligament is penetrated and the local anesthetic can be injected. This method safely avoids inadvertent penetration of the fetlock joint, which is a risk when (c) inserting the needle distal to the button of the splint bones, as is done for a conventional low four-point nerve block (2). However, neither this nor the alternative technique (b) can currently be performed on the simulators as the splint bones are firmly attached to the cannon bone and the needle cannot be advanced between them. In a clinical setting, procedure (b) can also combined with a modified abaxial sesamoid nerve block to achieve an effect very similar to a conventional low four-point nerve block. In summary, the low four-point nerve block and its variations (a), (b), and (c) with regard to the metacarpal nerves should be taught and discussed with students during specific classes for the sake of completeness.

The practical performance after the interventions was tested in the form of an OSCE station, which enabled a high degree of objectivity. Besides, the use of the simulator made it possible to achieve greater comparability of the students' performances, as no distortion of the results could be caused by anatomical and other variances of the cadaver limbs. In addition, the developed simulators were subjectively evaluated by different groups, including equine veterinarians.

When interpreting the results of the intervention study with 5^th^-year students, it has to be noted that the extent to which the learning effect was influenced by the introduction and demonstration at the beginning of the training session using a cadaver limb, or what learning effect would have occurred without it was not investigated. This study was deliberately designed to provide a comparison between the simulator and a cadaver limb in the current standard exercise. The use of a simulator in the examination was also viewed critically. The use of cadaver limbs could have made the object more realistic. In addition, verification of correct puncturing could have been carried out more precisely if the students had been able to inject dye. The examiner could then have dissected the cadaver limb to determine whether the injected dye had hit the correct nerves in the correct place. For hygienic and logistical reasons, cadaver limbs were not used in this study for the examination.

In conclusion, the developed simulators are suitable as instructional tools for equine perineural anesthesia. The simulators will not replace cadaver limbs as a teaching aid, but will complement them.

The simulators will be optimized based on the evaluation results. In particular, synovial structures are to be added to S1 and the haptics of S2 is to be improved by using thicker skin. In addition, the integrated success control of the simulators will be equipped with auditory feedback in future. The feedback system on S2 will be expanded so that feedback on the puncture site is only given after the puncture has been made and an image appears at the same time on which the anesthetized areas can be recognized. It is planned to offer a course at the CSL so that all TiHo students are given the opportunity to practice perineural anesthesia on the simulators and repeat the exercise as often as they like. In addition, students in their clinical year will receive an exercise with the simulators as part of a 3-day training session at the CSL before their clinical rotation at the Clinic for Horses. The simulation practice is intended to better prepare the students for the actual practice with cadaver limbs at the Clinic for Horses. It would be interesting to investigate further at what point in time during the veterinary studies and to what extent it makes sense to use the simulators for teaching purposes in order to achieve the best possible supplement to the hands-on practice on the cadaver limb. For this purpose, it would certainly also be interesting to carry out a study with a stepwise training with the different instructional tools.

## Data Availability

The datasets presented in this article are not readily available because of data protection. Requests to access the datasets should be directed to Florian Geburek, florian.geburek@tiho-hannover.de.
