# Supplementary material for: Evaluation of equine perineural anesthesia simulators with integrated success control for veterinary education
Source: Front Vet Sci. 2025 Jan 9;11:1403794. doi: 10.3389/fvets.2024.1403794 (PMC11754189; doi:10.3389/fvets.2024.1403794)
Supplement: Supplementary file 1 [file Data_Sheet_1.pdf]

Document 1: Translation of the OSCE-checklist: Perineural anesthesia

	**What the examinee should do:**	**Not fulfilled**	**Partially fulfilled**	**Fulfilled**	**Points**
1.	Prepare all necessary materials before carrying out the exercise<br>(gloves, alcohol swabs, sterile compresses, 3x needle, 3x syringe, local anesthetic)				1
2.	Select the correct needles *(2x 23 G and 1x 22 G)*				1
3.	Select the correct local anesthetic *(Mepivacain)*				2
4.	Apply the correct amount of local anesthetic *(3x 1-3 mL)*				2
5.	<u>Announcement:</u> Lifting of the limb by an assistant				1
a)	Modified abaxial sesamoid nerve block, medial				
6.	Identify the correct puncture site<br>*Palpation of the vascular nerve bundle (medial palmar nerve) approximately 20 mm above the fetlock joint at the proximal edge of the medial proximal sesamoid bone, abaxial to the superficial and deep digital flexor tendon*				3
7.	Disinfect the puncture site with alcohol swabs				1
8.	Pull up a skin fold at the puncture site				2
9.	Use a 23 G needle				1
10.	Grip the needle exclusively at the needle hub				3
11.	Puncture with the needle without a syringe attached				2
12.	Puncture subcutaneous, medio-palmar and parallel to the vascular nerve bundle to distal				4
13.	Before the puncture the bevel of the needle points away from the skin				2

14.	Attach a syringe with local anesthetic (with fixed needle)				1
15.	Aspirate (with fixed needle)				2
16.	Inject the local anesthetic (with fixed needle)				2
17.	Remove the needle with the syringe attached or stop the syringe beforehand				1
18.	Safely dispose of the needle in a waste container				1
19.	Compress the puncture site with a sterile compress for about five seconds				2
20.	The clinical skills were performed in the correct order				3
b)	Modified abaxial sesamoid nerve block, lateral				
21.	Identify the correct puncture site<br>*Palpation of the vascular nerve bundle (lateral palmar nerve) approximately 20 mm above the fetlock joint at the proximal edge of the lateral proximal sesamoid bone, abaxial to the superficial and deep digital flexor tendon*				3
22.	Disinfect the puncture site with alcohol swabs				1
23.	Pull up a skin fold at the puncture site				2
24.	Use a 23 G needle				1
25.	Grip the needle exclusively at the needle hub				3
26.	Puncture with the needle without a syringe attached				2
27.	Puncture subcutaneous, latero-palmar and parallel to the vascular nerve bundle to distal				4
28.	Before the puncture the bevel of the needle points away from the skin				2
29.	Attach a syringe with local anesthetic (with fixed needle)				1
30.	Aspirate (with fixed needle)				2
31.	Inject the local anesthetic (with fixed needle)				2

32.	Remove the needle with the syringe attached or stop the syringe beforehand				1
33.	Safely dispose of the needle in a waste container				1
34.	Compress the puncture site with a sterile compress for about five seconds				2
35.	The clinical skills were performed in the correct order				3
c)	Palmar metacarpal nerve block				
36.	Identify the correct puncture site<br>*Lateral approximately 3-4 cm proximal to the distal end of the lateral splint bone or halfway along the metacarpus, between the suspensory ligament and lateral splint bone*				3
37.	Disinfect the puncture site with alcohol swabs				1
38.	Use a 22 G needle				1
39.	Grip the needle exclusively at the needle hub				3
40.	Puncture with the needle without a syringe attached				2
41.	Axial displacement of suspensory ligament using the thumb				2
42.	Puncture at a 90° angle to the limb axis in front of the thumb				4
43.	Advance the needle until the tip is approximately centred on the cannon bone (about 1.5 cm)				2
44.	Attach a syringe with local anesthetic (with fixed needle)				1
45.	Aspirate (with fixed needle)				2
46.	Inject the local anesthetic (with fixed needle)				2
47.	Remove the needle with the syringe attached or stop the syringe beforehand				1
48.	Safely dispose of the needle in a waste container				1
49.	Compress the puncture site with a sterile compress for about five seconds				2

50.	The clinical skills were performed in the correct order				3
51.	The Palmar metacarpal nerve block was performed with only one puncture from the side				3
	General aspects				
52.	All three punctures: section a) - c) were performed (any order of sections a) - c)				4
53.	Aseptic procedures were followed for all skills				3
	**Total**				107

Document 2: Translation of the questionnaire on experience and self-efficacy

Questionnaire on self-efficacy

Please tick the answer that applies the most to you. Unless otherwise indicated, the answer options are single choices.

01 | Demographics

Semester: _____

Have you already completed a professional training or worked in a field in which perineural anesthesia was performed on horses prior to your studies?

☐ Yes ☐ No

02 | Practical experience

1. **How often have you performed perineural anesthesia on a live horse?** (an examination of a limb with one or more punctures = 1x)
   ☐ Not at all ☐ 1-5x ☐ 6-10x ☐ 11-20x ☐ > 20x

2. **How often have you performed perineural anesthesia on a cadaver limb?** (an examination of a limb with one or more punctures = 1x)
   ☐ Not at all ☐ 1-5x ☐ 6-10x ☐ 11-20x ☐ > 20x

3. **How often have you performed perineural anesthesia on a simulator?** (an examination of a limb with one or more punctures = 1x)
   ☐ Not at all ☐ 1-5x ☐ 6-10x ☐ 11-20x ☐ > 20x

4. **Have you completed internships in which perineural anesthesia was performed on horses? If so, what were you allowed to do?** (multiple answer options are possible)
   ☐ No ☐ Yes, watch ☐ Yes, prepare puncture site ☐ Yes, assist
   ☐ Yes, perform the perineural anesthesia yourself under supervision
   ☐ Yes, perform the perineural anesthesia independently

03 | Theoretical knowledge		Does not apply	Somewhat does not apply	Partially applies	Somewhat does apply	Fully applies
1.	I have extensive theoretical knowledge regarding the anatomy of an equine limb					
2.	I know the course and location of the relevant nerves of an equine limb for performing perineural anesthesia.					

3.	I know how to correctly perform perineural anesthesia on an equine limb (preparation, puncture, aftercare, assessment).					
4.	I know the puncture sites for perineural anesthesia on the forelimb of a horse.					
5.	I know the puncture sites for perineural anesthesia on the hindlimb of a horse.					
6.	I know which anatomical areas should be anaesthetized after the various perineural anesthesia have been performed correctly.					

**04 | Practical skills**		Does not apply	Somewhat does not apply	Partially applies	Somewhat does apply	Fully applies
1.	I am confident that I can palpate important anatomical structures for performing perineural anesthesia on an equine limb.					
2.	I am confident that I can correctly locate the puncture sites for perineural anesthesia on the forelimb of a horse up to and including high four-point nerve block.					
3.	I am confident that I can correctly locate the puncture sites of all perineural anesthesia on the forelimb of a horse on my own.					
4.	I am confident that I can correctly perform perineural anesthesia on the forelimb of a horse up to and including high four-point nerve block.					
5.	I am confident that I can perform all perineural anesthesia on the forelimb of a horse correctly and independently.					
6.	I am confident that I can perform all perineural anesthesia on the hind limb of a horse correctly and independently.					

Thank you for participating!
